# Posing as Ascites: A Case Report on Neurogenic Lower Urinary Tract Dysfunction

**DOI:** 10.7759/cureus.35829

**Published:** 2023-03-06

**Authors:** Luís Carlos Sousa, Maria João Andrade

**Affiliations:** 1 Physical Medicine and Rehabilitation, Centro Hospitalar Universitário do Porto, Porto, PRT; 2 Ensino Pré Graduado, Instituto de Ciências Biomédicas Abel Salazar - Centro Hospitalar Universitário do Porto, Porto, PRT

**Keywords:** spinal cord injury, overflow incontinence, urinary retention, neuro-urological examination, neurogenic lower urinary tract dysfunction

## Abstract

Lower urinary tract dysfunction (LUTD) is a frequently neglected and underdiagnosed condition, especially in cases of neurogenic etiology where no other neurological deficits are present. The evaluation of the integrity of the spinal cord segments responsible for the neurophysiologic control of the bladder and sphincters is fundamental for correctly establishing a neurogenic etiology.

We present the case of a 52-year-old female complaining of abdominal pain, new onset of urinary straining, a slow/intermittent stream, and stress incontinence, following inpatient admission for a history of constitutional syndrome and falls. A fluid wave sign was observed on physical examination. An abdominal CT scan showed bladder hyperdistention and an L5 body compression fracture. A urinary catheter was placed, draining 2,000 mL of urine. On neuro-urological examination, diminished anal sphincter tone, diminished voluntary anal contraction, and absent left anal reflex were noted. Findings on the urodynamic study further favored the diagnosis of lower motor neuron bladder dysfunction.

This case report demonstrates how the neurologic examination of the sacral segments S2-S4 allowed the diagnosis and subsequent management of an initially unexplained bladder dysfunction, as our clinical findings were compatible with damage to the sacral roots. The complete neuro-urological examination is fundamental for correctly determining the neurogenic etiology of LUTD and should be routinely integrated into the neurological evaluation.

## Introduction

Lower urinary tract dysfunction (LUTD) is a frequently neglected and underdiagnosed condition, especially in cases of neurogenic etiology where no other neurological deficits are present. The evaluation of the integrity of the spinal cord segments responsible for the neurophysiologic control of the bladder and sphincters, namely, thoracolumbar T11-L2 and sacral S2-S4 segments, is fundamental for correctly establishing a neurogenic etiology. In our clinical practice, we accomplish this by examining anal sensory function, voluntary anal contraction, anal tone, and the sacral and thoracolumbar reflexes, including bulbocavernosus (male), clitoroanal (female), anal, cremasteric, and Dartos reflexes [1].

This article was previously presented as a meeting poster at the 13th International Society of Physical and Rehabilitation Medicine World Congress on June 9-13, 2019.

## Case presentation

A 52-year-old female was admitted with a week-long history of diarrhea, fever, and constitutional symptoms (anorexia, malaise, and weight loss). She had a history of HIV infection and cerebral toxoplasmosis with left-sided spastic hemiparesis and cognitive impairment as sequelae. She was autonomous in the activities of daily living and used a crutch for walking. She had no prior complaints of LUTD.

She reported falling three times, with consequent trauma to the head, lower back, and lower limbs. An imaging workup obtained on admission, including a plain radiograph of the lumbosacral spine, revealed a compression fracture of the L5 vertebra, which was overlooked in the emergency department because at the time there was no evidence of new neurologic deficits. The rest of the imaging studies, namely, a brain CT scan and plain radiographs of the patient’s hips and knees, showed no evidence of trauma or new lesions of any etiology. The patient presented with fever (38.5°C), hypotension (blood pressure 88/44 mmHg), tachycardia (107 beats/minute), hypoglycemia (33 mg/dL), and normal SpO_2_. Her bloodwork showed pancytopenia, elevated C-reactive protein, elevated liver enzymes, and hypoalbuminemia. Blood cultures revealed the growth of Gram-negative bacteria.

**Figure 1:**
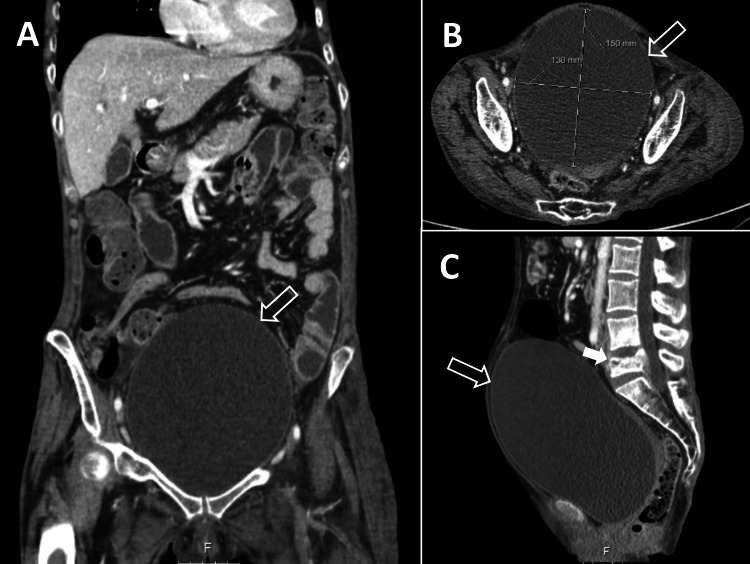
Abdominal CT scan. A: coronal view showing a hyperdistended bladder (void arrows); B: horizontal view with bladder measurements; C: sagittal view showing bladder hyperdistention and L5 compression fracture (white arrow).

Following inpatient admission to the Infectious Diseases ward for sepsis secondary to infectious diarrhea, over the course of three weeks, she developed diffuse abdominal pain with a recurrent pattern, abdominal distension, and a positive fluid wave test on abdominal examination. Concurrently, she reported a new onset of urinary straining, a slow/intermittent stream, and stress incontinence (which worsened during the night). She also mentioned lumbar pain with irradiation through the left lower limb, which worsened with mobilization, and increased difficulty walking. The abdominal symptoms and signs led the infectiologists to investigate possible ascites. An abdominal CT scan was obtained on day 23 of inpatient admission (Figure 1). At the time, the motor and urinary complaints were not considered in the differential diagnosis of the abdominal symptoms and signs. The attending team attributed the urinary complaints to a possible urinary infection and pursued diagnosis, but urinalysis and urine culture were negative. Our consultation was solicited for functional rehabilitation in the context of impaired mobility.

The abdominal CT scan showed bladder hyperdistention, bilateral ureterohydronephrosis, and an L5 body compression fracture. The radiologist’s report suggested urinary catheterization. A urinary catheter was placed, draining 2,000 mL of urine.

We observed the patient at this time. On our evaluation, we acknowledged the patient’s motor and urinary complaints, which coupled with the imaging evidence of LUTD and a concurrent lumbar fracture led to the performance of a neurological examination that included the evaluation of the sacral segments. On our examination, we found diminished anal sphincter tone, diminished voluntary anal contraction, absent left anal reflex, preserved clitoroanal reflex, and preserved anal sensation (pinprick and deep anal pressure). The rest of the neurological examination revealed a previously known left-sided spastic hemiparesis with superimposed motor deficits in the lower limbs, with motor strength testing (right/left) according to the International Standards for the Neurologic Classification of Spinal Cord Injury (ISNCSCI), L2 3/1, L3 4/1, L4 4/2, L5 4/3, S1 3/2; it also showed diminished Achilles reflex on the left and absent on the right. Inconsistent responses on sensory examination due to cognitive impairment limited our ability to ascertain the presence of a sensory level.

A urodynamic study was performed on day 46 to characterize LUTD. It revealed normal bladder sensation, absent voluntary detrusor contraction, inefficient micturition with a low-flow pattern, and elevated residual volumes (240-350 mL).

**Figure 2:**
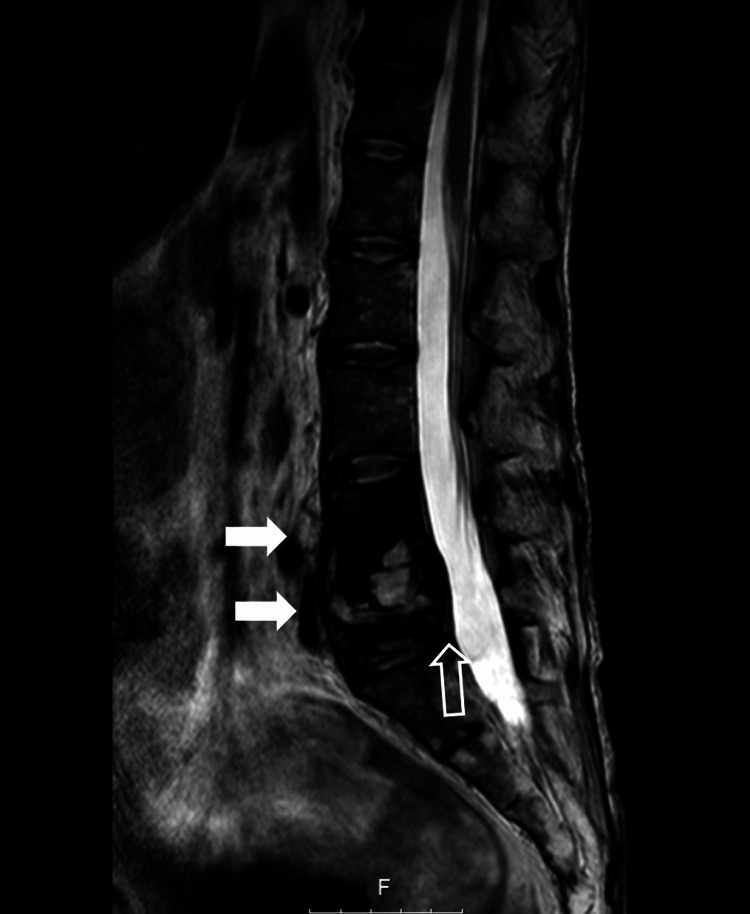
Lumbosacral MRI. Lumbosacral MRI showing posterior displacement of L5 (void arrow) and inflammatory changes in L4, L5, and paravertebral tissues (white arrows).

To further investigate these findings, a lumbosacral MRI scan was obtained one day later. It showed posterior displacement of the body of L5 contacting the thecal sac, as well as inflammatory changes in L4, L5, and paravertebral tissues. The MRI report had no mention of structural damage to the neural elements of the spinal canal, namely, of cauda equina root involvement (Figure 2).

Following the investigation, the patient started intermittent catheterization with a previous attempt to void and an α-blocker (tamsulosin). She resumed spontaneous micturition with negligible residual volumes approximately one month later.

## Discussion

Neurophysiological control of bladder and sphincter function is a complex system involving multiple structures of the central and peripheral nervous systems. The frontal cortex, the periaqueductal gray matter, and the pontine micturition center are the primary brain centers responsible for volitional control of micturition and coordination of the detrusor and urethral sphincter function (contraction/relaxation). The micturition reflex is a medullary reflex mediated by parasympathetic and somatic innervation involving the sacral cord segments S2-S4 and sympathetic innervation of the thoracolumbar segments T11-L2. The peripheral innervation of the bladder and urethral sphincters (internal and external) is mediated by three sets of nerves, namely, hypogastric, pelvic, and pudendal nerves, which include afferent and efferent fibers intervening in the vesicosphincteric function [2]. The integrity of all structures is critical for the physiological storage and voiding functions of the bladder. As such, depending on the site of the lesion, neurogenic lower urinary tract dysfunction (NLUTD) may have different clinical presentations, affecting the storage and/or emptying function [1].

The neurological examination may allow the identification of the site of the lesion responsible for NLUTD [3]. This is particularly important in cases of unexplained bladder dysfunction in the absence of a known neurological problem, as the neurogenic etiology may not be evident from the initial evaluation. To test the sacral cord segments (S2-S5), the evaluation must include deep anal pressure, perianal sensation, anal tone, voluntary anal contraction, and the sacral reflexes, including bulbocavernosus/clitoroanal (male/female) and anal reflexes [1,4].

In this clinical case, the patient’s complaints about urinary straining coupled with the imaging evidence of urinary retention were compatible with LUTD [2]. The reports of stress incontinence were, in this case, consistent with overflow voiding. Overflow incontinence and inability to empty the bladder characterize a lower motor neuron injury [1], supporting the hypothesis of damage to the sacral cord segments and/or cauda equina roots S2-S4. In this case, the presence of lumbar trauma with a fractured L5 vertebra favored a lesion to the sacral roots as the most likely cause of urinary dysfunction [5].

The only way to clinically evaluate function in these segments was by performing the examination of the sacral segments, as mentioned above. All findings on the sacral examination (absent unilateral anal reflex and diminished anal contraction and tone) pointed to functional impairment of the sacral segments S2-S4, thus corroborating the history and imaging findings.

The urodynamic results showed further evidence of LUTD. Damage to the small autonomic postganglionic nerve fibers innervating the detrusor or the sensory suburothelial plexus secondary to bladder hyperdistention cannot be excluded and may have played a role in the development of NLUTD. The evidence of normal bladder sensation supports the integrity of the myelinated afferent fibers (A-delta) conveying the sensation of bladder fullness, showing that at least partial innervation of the lower urinary tract was preserved [1].

At the time the MRI was obtained, there was no evidence of structural impairment of the cauda equina roots consequent to the trauma or the inflammatory changes. However, there was substantial clinical and urodynamic evidence of NLUTD pointing to functional impairment of these structures. The trauma and consequent inflammation of the vertebral and paravertebral structures may have resulted in transient damage to the cauda equina roots which was not apparent at the time the MRI was obtained. Therefore, the absence of radiologic evidence of structural impairment does not exclude functional impairment.

## Conclusions

The findings of the neuro-urological examination confirmed the injury of the sacral roots, which explains the episode of bladder dysfunction. The neurological examination of the sacral cord segments was fundamental for correctly determining the neurogenic etiology of initially unexplained bladder dysfunction and orienting subsequent investigation and treatment.

The complete neuro-urological examination should be routinely integrated into the neurological evaluation because it allows testing somatic, sympathetic, and parasympathetic innervation controlling function of the lower urinary tract.

## References

[1] Panicker JN, Kalsi V, de Sèze M (2010). Approach and evaluation of neurogenic bladder dysfunction. Pelvic Organ Dysfunction in Neurological Disease: Clinical Management and Rehabilitation.

[2] Fowler CJ, Griffiths D, de Groat WC (2008). The neural control of micturition. Nat Rev Neurosci.

[3] Wyndaele JJ (1992). Neurourology in spinal cord injured patients. Paraplegia.

[4] Rushton DN (1994). Neuro-urological history and examination. Handbook of Neuro-Urology.

[5] Eisen A (2019). Anatomy and localization of spinal cord disorders. UpToDate.

